# Posture evaluation and treatment in adolescents through wearable technology: a systematic review

**DOI:** 10.3389/fresc.2026.1587466

**Published:** 2026-03-18

**Authors:** Sara Liguori, Antimo Moretti, Viviana Andreozzi, Claudio Catalano, Gabriele Pontillo, Luca Maresca, Michele Riccio, Giovanni Breglio, Marco Paoletta, Francesca Gimigliano, Giovanni Iolascon

**Affiliations:** 1 Department of Medical and Surgical Specialties and Dentistry, University of Campania “Luigi Vanvitelli”, Naples, Italy; 2 Department of Mental and Physical Health and Preventive Medicine, University of Campania “Luigi Vanvitelli”, Naples, Italy; 3 Department of Architecture, University of Florence, Florence, Italy; 4 Department of Electrical Engineering and Information Technologies, University of Naples “Federico II”, Naples, Italy

**Keywords:** adolescent, digital health, posture, spine, wearable device

## Abstract

**Introduction:**

Postural health is crucial during adolescence, a period marked by rapid physical growth and increased susceptibility to postural deformities due to sedentary lifestyles. Wearable technologies offer a promising solution for spinal posture monitoring and correction, utilizing sensors, inertial measurement units (IMUs), and artificial intelligence to provide real-time biofeedback. However, current wearable devices face limitations, including inconsistent methodologies, sensor attachment issues, and the absence of immediate corrective feedback. This systematic review aims to determine the status of clinical and experimental research in the area of wearable technology designed for spinal monitoring in adolescents.

**Methods:**

Following PRISMA guidelines, we searched multiple databases for relevant studies published until December 31, 2024, identifying studies about technology/ies able to monitor posture of the spine in the adolescents. The Systematic review protocol is available in the International Prospective Register of Systematic Reviews (PROSPERO) with the following number: CRD42024532328.

**Results:**

Three studies have been identified including a total of 103 participants, comprising 58 males and 45 females Findings indicate that while existing wearable systems can track posture, they often lack methodological rigor, real-time feedback capabilities, and comprehensive evaluation protocols.

**Discussion:**

There is a critical need for innovative digital health solutions that integrate real-time monitoring and personalized interventions to address postural issues effectively. Despite their potential, wearable technologies face challenges related to usability, effectiveness, and evidence quality. Future research should focus on developing user-friendly designs, strengthening methodological approaches, and integrating multiple monitoring techniques to enhance spinal health in adolescents.

**Systematic Review Registration:**

https://www.crd.york.ac.uk/PROSPERO/recorddashboard, PROSPERO CRD42024532328.

## 1 Introduction

Posture is a “habitual attitude” of an individual determined by the contraction of groups of skeletal muscles that ensure a certain position of the body and its parts in space (1).

Posture is considered correct when it reflects an ideal musculoskeletal interaction, representing the most favorable state in terms of ergonomic outcomes and physio-anatomical alignment of structural components (2). When this ideal state is not achieved, functional and symptomatic consequences may arise.

This issue is particularly common among adolescents, especially in industrialized countries, due to sedentary habits and significant changes in physical and mental development. These factors may lead to spinal postural alterations in both the sagittal and frontal planes (3). Yang et al. reported that 34%–50% of adolescents had different degrees of incorrect posture with a higher prevalence in girls (3).

To date, the diagnosis and management of poor posture is characterized by a lack of standardized evaluation protocols and convergence of methods (4–6).

Currently, the clinical methods to assess spinal alignment in adolescents range from observational approaches, such as standing posture assessment and the Adams forward bend test, to instrument-assisted techniques including plumb line assessment and scoliometer measurements. Radiographic imaging, particularly standing x-rays with Cobb angle measurement, remains the gold standard for definitive diagnosis and longitudinal monitoring of spinal curvature (7–10). Regarding the management of poor posture and spinal deformities in adolescents, there are currently no fully standardized treatment protocols supported by high-quality evidence across all clinical contexts. Preventive and conservative strategies primarily aim to improve postural control, limit curve progression, and delay or avoid invasive interventions. Physiotherapeutic approaches such as Global Postural Re-education (GPR) focus on muscle chain rebalancing, postural alignment, and breathing control, and may improve flexibility and balance in adolescents with postural alterations (11). Similarly, Physiotherapeutic Scoliosis-Specific Exercises (PSSE)-including methods such as the Schroth approach-are designed to enhance postural awareness, promote three-dimensional self-correction, and reduce the risk of curve progression in mild to moderate scoliosis. These interventions are recommended by international guidelines (e.g., SOSORT) but require high patient adherence and trained therapists, which may limit their effectiveness in real-world settings (12). Despite the availability of these clinical and therapeutic approaches, their effectiveness in everyday settings is often constrained by limited standardization, variable adherence, and the need for specialized supervision (27). In recent years, this gap has stimulated growing interest in technology-based solutions. Various wearable systems have been designed capable of detecting spinal posture and providing real-time biofeedback when poor posture is sustained (13). It is hypothesized that prolonged use of these wearables may lead to improved spinal posture over time. Wearable devices incorporate diverse technologies worn on the body, capable of measuring parameters such as step count, distance traveled, heart rate, and sleep quality, among others (14–17). Wearable devices incorporate diverse technologies worn on the body, capable of measuring parameters such as step count, distance traveled, heart rate, and sleep quality, among others (14–17). In this scenario, wearable systems specifically designed to monitor posture and provide corrective feedback are increasingly being developed across different contexts, including the workplace (18). These emerging tools offer continuous, real-world monitoring that traditional clinical assessments cannot provide, making them particularly relevant for adolescents whose postural habits evolve throughout daily activities. As research advances, understanding how these devices collect, process, and exchange posture-related data becomes essential for improving early detection, supporting preventive strategies, and enhancing the effectiveness of conservative interventions. The aim of this review is to examine available wearable systems designed to collect, process, and exchange posture-related data, with the goal of enhancing understanding of spinal postural patterns in children and adolescents and ultimately informing improved prevention and treatment approaches.

## 2 Materials and methods

### 2.1 Search strategy

According to the guidelines of the Preferred Reporting Items for Systematic Reviews and Meta-Analyses (PRISMA) (19), we conducted a systematic review by searching three databases (PubMed, EMBASE, and SCOPUS) for articles published from the inception until December 31, 2024. We followed the specific thesaurus for each database and utilized the strategy outlined in Table 1.

**Table 1:** Search strategy.

PubMed
(“Wearable Electronic Devices”[Mesh] OR “Digital Health”[Mesh] OR “Mobile Applications”) AND (“Posture”[Mesh]) AND (“Adolescent”[Mesh])
Embase
‘mobile application’ OR ‘wearable computer’ OR ‘digital health’ AND adolescent AND ‘posture control’ OR ‘posture correction brace’
Scopus
TITLE-ABS-KEY (“SMARTPHONE APPLICATION” OR “WEARABLE ELECTRONIC DEVICE” OR “DIGITAL HEALTH”) AND (“adolescent”) AND (“posture”)

The Systematic review protocol is available in the International Prospective Register of Systematic Reviews (PROSPERO) with the following number: CRD42024532328. The final choice of key search terms was derived from pre-established headings on the OVID Medline database after a generic screening using a list of relevant key terms.

Key search terms included: “Wearable Electronic Devices” OR “Digital Health” AND “Posture” AND “Adolescent”. Relevant MeSH (Medical Subject Heading) terms, spelling variations and synonyms were included and modified as appropriate for each database. Studies addressing both wearable systems and posture in adolescents were selected.

### 2.2 Selection criteria

Inclusion and exclusion criteria have been documented in Table 2. Duplicate studies were eliminated, with preference given to journal papers over conference papers. If multiple studies utilized the same dataset, the most recent update was considered. Initial screening was carried out by an independent reviewer (SL) who assessed selected articles based on the established criteria. A secondary screening was then completed by another independent reviewer (VA), following the same criteria. Any disagreement was solved by a third reviewer's (MP) consultation.

**Table 2:** Inclusion and exclusion criteria.

Inclusion criteria
1. Technology/ies (including apps and software tools, smartphone apps and mobile application tools) able to monitor posture of the spine in the adolescents
2. Technology/ies (including apps and software tools, smartphone apps and mobile application tools) able to monitor posture in the sagittal and/or coronal planes
3. Articles written in English
4. Time of publication from the inception to 31 December 2024
Exclusion criteria
1. Technology/ies (including apps and software tools, smartphone apps and mobile application tools) capable of identifying or discriminating between body positions (e.g., sitting, standing lying)
2. Technology/ies (including apps and software apps tools, smartphone apps and mobile application tools) capable of monitoring posture of body parts other than the spine
3. Wearable technology/ies classed as robotic or exoskeletons
4. Adult participants aged 18 years and older
5. Systematic reviews, book's chapter
6. Studies conducted on animals

### 2.3 Data extraction and quality assessment

In accordance with PRISMA guidelines for Quasi-Experimental Studies the selection process employed independent reviewers and validated bias assessment tools (PEDro scale and Joanna Briggs Institute Critical Appraisal Checklist for Quasi-Experimental Studies) to evaluate the included studies. After selecting articles, two researchers (SL and VA) collected data according to a predetermined checklist including participants, intervention, outcome measures, and main findings, using a customized Microsoft Excel sheet; any disagreements were resolved through a third reviewer (CC). The extracted data included (1) First author; (2) Journal; (3) Publication year; (4) Study design; (5) Age of study participants; (6) Sex of study participants; (7) Main characteristics of study participants.

## 3 Results

**Figure 1:**
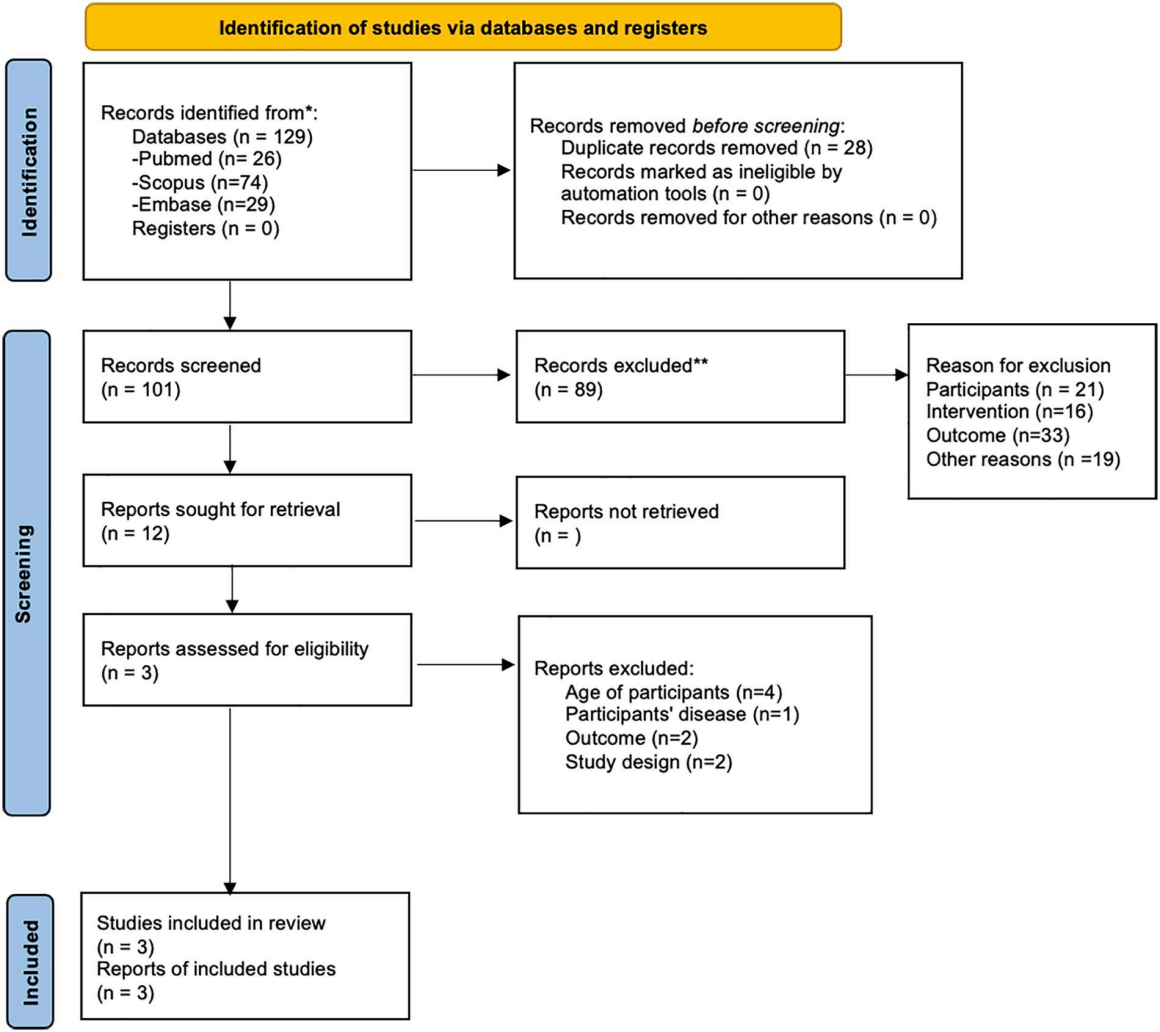
PRISMA 2020 flow diagram for new systematic reviews which included searches of databases and registers only. *Consider, if feasible to do so, reporting the number of records identified from each database or register searched (rather than the total number across all databases/registers). **If automation tools were used, indicate how many records were excluded by a human and how many were excluded by automation tools.

The PRISMA flow chart is illustrated in Figure 1. Out of 129 records identified from the databases, 89 were selected for abstract screening after removing 28 duplicate records. Abstract screening excluded 21 records due to the age of participants not meeting the pre-established inclusion criteria. Sixteen records were excluded because the interventions did not utilize technologies (including apps, software tools, smartphone apps, and mobile application tools) capable of monitoring posture in the sagittal and/or coronal planes or technologies for monitoring spinal posture in adolescents. Additionally, 33 articles were excluded because they did not evaluate posture, and another 19 articles were excluded for not meeting the other inclusion criteria.

The remaining 12 reports underwent full-text screening, which resulted in the exclusion of 9 reports for reasons detailed in the flow chart in Figure 1. Overall, 3 reports were assessed for eligibility, and their main findings are summarized in Table 3.

**Table 3:** Characteristics of the included studies.

Author, Year	Study design	Sample size: Total (Group)	Intervention	Outcome	Main findings
Gal-Nadasan et al., 2017 (20)	Observational study	*N* = 30 adolescents (18 M; 12 F) (16 years old) with incorrect posture due to incorrect sitting position in school benches.	Students were measured with a non-invasive, non-radiant and marker less human body tracking method based on the Microsoft Kinect 3D sensor compared to a physical exam by a medical rehabilitation specialist.	Diagnosis of scoliosis Y/N Microsoft Kinect 3D further explored: difference between the two shoulder's heights; angle between the two shoulders and the neck; rotation of the shoulders; difference between the two hip's heights; angle created by the two hips at the center of the hip; rotation of the hips	Most of the students have a higher shoulder height difference value than expected. The confusion matrix confirms that the Kinect device can be used in the high school education system to monitor the changes of the spinal cord to detect in early stages the signs of scoliosis and kyphosis.
Cheung et al., 2023 (21)	Qualitative, non-RCT study	*N* = 13 adolescents (6F; 7M) (11–13 years old) with mild scoliosis (15.2° ± 1.6°)	30 training sessions of sEMG biofeedback posture training program, once a week for 8 months. Each session lasted approximately 60 min, with adolescents instructed to sit in an ideal posture and relax four pairs of paraspinal muscles for 5 min, five times in each session with a 2-minute rest in between. Visual feedback appeared on the biofeedback screen to indicate muscle activities and the muscle activity ratio between the left and right sides.	Change in Cobb angle assessed by x-ray Benefits on the health-related quality of life through in-depth interviews	Cobb angles either decreased by at least 5° (*n* = 5) or showed minimal curve progression, with changes in the Cobb angle controlled under 5° (*n* = 8) at the x-ray exam. Several subthemes related to the benefits of the training program on the health-related quality of life were generated, namely (a) posture correction, (b) improvement in body appearance, (c) restoration of muscle relaxation, (d) reduction in bodily pain and fatigue, (e) enhancement of self-confidence/self-image, and (f) improvement in social functioning.
Perimal et al., 2023 (22)	non-RCT study	*N* = 73 children of 12 ± 3.7 years. (46 M; 27 F).	Children played to Ocean Explorer, a mobile digital game where players explore the in-game ocean environments in a submersible vehicle. Gameplay was recorded using Kinovea, with a lateral view of the seated subject's sagittal plane. Accelerometer data is captured through an inbuilt function within the Ocean Explorer game during each child's play session. During these sessions, the device was held on its side in the landscape position.	Authors reported the real-world movement correlations with the three axial planes (x, y, and z) measured by the in-built iPad accelerometer were established: -*x*-axis (pitch): flexion forward or backward -*y*-axis(yaw): rotation of the body left or right -z-axis (roll) lateral flexion of the body left or right.	A positive trend was showed in -*x*-axis (from approximately −10 to 0; y = 0.0082x – 9.6569, R2 = 0.1003). -*y*-axis (from approximately 6 to 16; y = 0.0083x + 6.4784, R2 = 0.911). -z-axis exhibited a very slight negative trend from about 0 to −2 (y = −0.0008x – 0.0538, R2 = 0.0186).

RCT, randomized controlled trial; SAPO, software de avaliação postural; sEMG, surface electromyography.

### 3.1 Characteristics of included studies

A total of 103 participants were included, comprising 58 males and 45 females. One study reported an age range of 11 to 13 years, one study indicated a mean age of 16 years without providing further details, and another study reported a mean age of 12 years (SD ± 3.7). Each study investigated a distinct technological approach:

- One study focused on posture evaluation using a digital assessment tool (20).
- One study evaluated a rehabilitation-oriented system incorporating biofeedback mechanisms (21).
- One study assessed a mixed-use wearable system designed for both monitoring and intervention purposes (22).

Only one study reported a clearly defined primary outcome, namely radiographic assessment of Cobb's angle in adolescents with mild scoliosis (21). The remaining studies relied on indirect or technology-derived postural metrics, with limited clinical outcome validation (20, 22).

### 3.2 Methodological quality and risk of bias

Methodological quality was assessed using the Joanna Briggs Institute Critical Appraisal Checklist for Quasi-Experimental Studies (non-randomized experimental studies). Risk of bias was evaluated across nine domains and is summarized in Table 4. All included studies were non-randomized, automatically leading to a lower certainty of evidence per GRADE criteria. Recurring methodological limitations included:

- Small sample sizes, reducing statistical power and limiting external validity.
- Absence of control groups or pre-post design in some cases, weakening causal inference.
- Insufficient reporting on blinding and confounder management, affecting internal validity.
- Device validation procedures and protocol descriptions were often vague, limiting reproducibility and generalizability.

**Table 4:** Joanna Briggs Institute critical appraisal checklist for quasi-experimental studies (non-randomized experimental studies).

Author and Year	Q1	Q2	Q3	Q4	Q5	Q6	Q7	Q8	Q9
Gal-Nadasan et al., 2017 (20)	U	Y	Y	U	N/A	N/A	Y	Y	U
Cheung et al., 2023 (21)	Y	Y	Y	N	Y	N/A	Y	Y	Y
Perimal et al., 2023 (22)	U	Y	Y	N	N/A	N/A	Y	Y	Y

Q1=Is it clear in the study what is the “cause” and what is the “effect” (i.e., there is no confusion about which variable comes first)?; Q2=Were the participants included in any comparisons similar?; Q3=Were the participants includes in any comparisons receiving similar treatment/care, other than the exposure or intervention of interest?; Q4=Was there a control group?; Q5=Were there multiple measurement of the outcome both pre and post the intervention/ exposure?; Q6=Was follow-up complete, and if not, were differences between groups in terms of their follow up adequately described and analyzed?; Q7=Were the outcomes of participants included in any comparisons measured in the same way?; Q8=Were outcomes measured in a reliable way?; Q9=Was appropriate statistical analysis used?; N, no; Y, yes; N/A, not applicable.

### 3.3 Data synthesis considerations

Due to considerable heterogeneity in study design, target populations, technologies used, and outcomes measured, a quantitative meta-analysis was not feasible. Pooling data under these conditions was considered methodologically inappropriate and potentially misleading. Although inter-rater reliability was ensured via consensus with a third reviewer, the lack of standardization across data sources and outcomes reflects a broader issue in the field: the absence of unified protocols and consensus-based metrics.

## 4 Discussion

**Figure 2:**
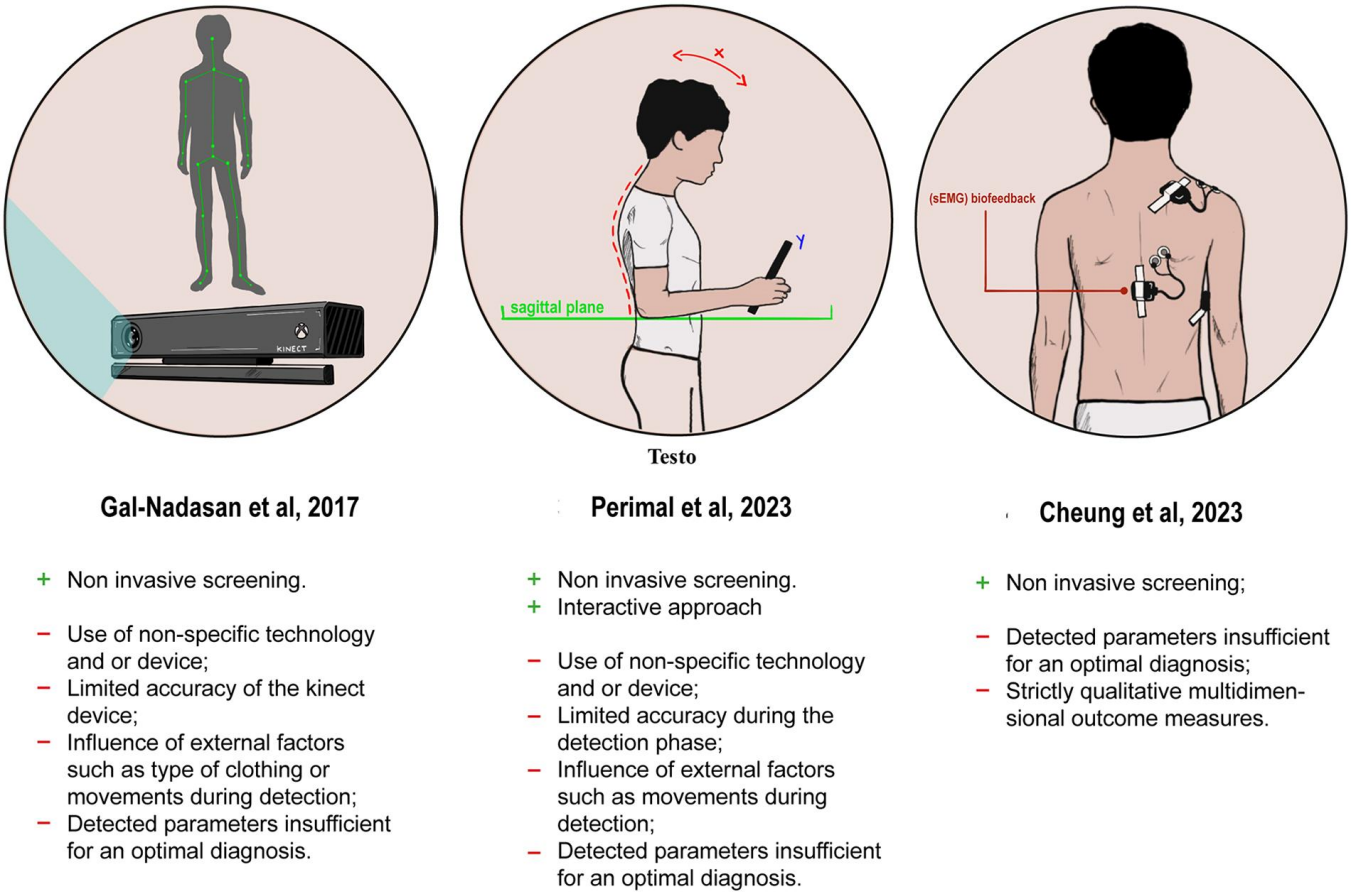
Pros & cons of the available wearable devices used to monitor poor posture in adolescents.

Adolescence is a critical period for health and development, focusing on self-image, emotional management, and physiological changes like body posture. Proper posture is characterized by the alignment of body segments unaffected by pathology and is influenced by physiological, biomechanical, occupational, and behavioral factors (2). Disruptions during this period may lead to persistent postural deviations with functional consequences later in life. For this reason, early detection and monitoring of posture in adolescents is a clinically relevant goal. Overall, the present review highlights that currently available wearable and digital systems for posture monitoring in adolescents remain methodologically fragmented. Across studies, there is no convergence toward standardized assessment protocols, outcome measures, or validation strategies. This lack of uniformity limits comparability between devices and weakens the evidence supporting their clinical and therapeutic utility. Figure 2 summarizes the main strengths and weaknesses of the three devices proposed.

Vision-based systems, such as those employing Microsoft Kinect, represent one of the earliest digital approaches to postural assessment in adolescents. Gal-Nadasan et al. (20) demonstrated the feasibility of capturing kinematic postural data in ecological environments, such as schools, allowing observation of posture during daily activities. These systems can generate large datasets and provide a longitudinal overview of postural behavior. However, their clinical applicability is constrained by several factors. Accurate monitoring requires participants to remain within a defined visual field, limiting use in real-world settings. Moreover, data analysis is performed *post hoc*, and no real-time feedback is provided to facilitate immediate posture correction. As a result, while vision-based systems are valuable for observational research, they offer limited support for active intervention or continuous monitoring. More advanced prototypes attempt to integrate multiple sensing modalities or embed monitoring capabilities into therapeutic devices. Cheung et al. employed surface electromyography (sEMG) to detect movement asymmetries, but the absence of concurrent kinematic data limited interpretation of muscle activation patterns (21). Combining sEMG with IMU-based motion tracking could provide a more comprehensive representation of posture by linking muscle activity to movement. In this scenario, Xuan et al. proposed a smart spinal orthosis incorporating flexible pressure sensors for real-time monitoring in adolescents with idiopathic scoliosis (23). This system represents an important step toward personalized, adaptive treatment, incorporating compensation for environmental factors such as temperature and humidity. However, despite its technological sophistication, the prototype lacked user-centered design features, intuitive interfaces, and direct patient feedback mechanisms, all of which are critical for adolescent compliance (23). An interesting approach in the literature was proposed by Moreira et al., who used biophotogrammetric techniques and machine learning to accurately estimate anatomical landmarks and extract essential data for postural assessment (24). In this context, wearable devices based on IMUs represent a more portable and potentially scalable solution. Nevertheless, most IMU-based devices currently rely on offline data processing, providing feedback only after acquisition. This temporal delay limits their corrective potential. The integration of onboard artificial intelligence could overcome this limitation by enabling real-time processing of IMU data and immediate user feedback. Such an approach may enhance adherence and promote active postural self-correction, but remains insufficiently explored in adolescent populations.

Overall, these studies provide valuable insights into wearable technology for physical well-being but highlight the need for broader participant inclusivity and more comprehensive methodologies in future research.

One of the challenges is the lower compliance of adolescents in recruitment and in treatment, particularly when treatments require sustained effort over time (25). The heterogeneity within this age group—variations in developmental stages, levels of physical activity, and attitudes towards health—further complicates recruitment and adherence to treatment protocols. Monitoring the progression of postural deformities is also challenging, as these conditions can evolve rapidly during adolescence (26). Additionally, the limited number of published studies on this topic often fails to meet rigorous quality standards. Many lack the methodological robustness required to produce reliable data, complicating our understanding of effective interventions for this population. Consequently, there is a pressing need for further research to explore innovative solutions—particularly the integration of digital health technologies that provide tailored monitoring and treatment options for adolescents with poor posture. The present review identifies several gaps that limit the strength of current evidence. The scarcity of rigorous experimental designs, together with the absence of long-term follow-up data, reduces confidence in the reported outcomes. Moreover, the lack of validated tools specifically tailored to adolescent posture monitoring restricts the comparability of findings across studies. These limitations underscore the need for future research to adopt more robust methodological frameworks and to develop standardized, age-appropriate assessment tools capable of supporting reliable evaluation and intervention strategies.

## 5 Conclusion

The prevalence of incorrect posture among adolescents poses significant health risks, including chronic spinal pain and deformities. Wearable technologies offer a promising approach for monitoring and correcting posture, yet many existing systems often lack methodological rigor and real-time user feedback. Enhancing these devices will require the integration of real-time data processing and user-centered design principles. Furthermore, further research is crucial to establish strong evidence supporting the effectiveness of wearable technologies in improving spinal posture among adolescents. Addressing these gaps will enable the development of more effective preventive measures and treatment strategies, ultimately promoting better musculoskeletal health during this critical developmental stage.

## Data Availability

The raw data supporting the conclusions of this article will be made available by the authors, without undue reservation.
